# Microwave Synthesis in Zeolite and MOF Membranes

**DOI:** 10.3390/membranes15090275

**Published:** 2025-09-12

**Authors:** Liangqing Li

**Affiliations:** 1 Key Laboratory of Functional Membranes and Energy Materials, School of Chemistry and Chemical Engineering, Huangshan University, Huangshan 245041, China; liliangqing@hsu.edu.cn; 2 School of Chemistry and Materials, University of Science and Technology of China, Hefei 230026, China

**Keywords:** zeolite membrane, metal–organic framework membrane, microwave synthesis, heating rate, single-mode microwave

## Abstract

Zeolites and metal–organic frameworks (MOFs) are crystalline porous materials characterized by highly ordered pore structures. Their fabrication into membranes has demonstrated significant potential for use in separation processes involving liquids or gases. Traditional methods for synthesizing these membranes often require prolonged reaction times and high energy input. In contrast, microwave heating technology has gained increasing attention as a more efficient approach for the synthesis of zeolite and MOF membranes, offering advantages such as rapid and uniform heating, enhanced energy efficiency, and greater environmental sustainability. This review focuses on fundamental research and laboratory-scale studies on the microwave-assisted synthesis of zeolite and MOF membranes. It begins by outlining the principles of microwave heating, emphasizing the mechanisms that enable accelerated heating. The discussion then highlights the key features and advantages of microwave synthesis in membrane fabrication, including reduced synthesis times, thinner membrane layers, suppression of impurities and undesired phases, and enhanced membrane density. Recent advancements in this area are also presented, particularly strategies for optimizing microwave heating processes, such as the use of single-mode microwave systems and precise control of heating rates. Notably, optimized microwave synthesis with controlled heating rates has been shown to reduce crystallization time by approximately 69%, decrease membrane thickness by nearly 70%, and improve pervaporation flux for acetic acid dehydration by more than 70%, compared with conventional microwave synthesis of mordenite membranes. Finally, the review summarizes and presents future perspectives aimed at promoting continued research and refinement of synthesis strategies in this promising area.

## 1. Introduction

Among various separation technologies, membrane separation has developed rapidly in recent years due to its simple operation, low energy consumption, and high separation efficiency [1,2,3,4,5,6]. It has been widely applied in areas such as organic solvent dehydration, seawater desalination, and gas separation [7,8,9,10,11]. Membrane materials play a crucial role in the performance of membrane separation processes [12]. Over the past few decades, a wide range of membranes have been developed and fabricated [13,14,15,16,17,18]. In particular, zeolite and MOF membranes have attracted significant attention because of their well-defined pore structures and exceptional molecular sieving capabilities. These properties enable precise molecular-level separation, making them highly promising for applications involving the separation of liquid or gas molecules [19,20,21,22,23,24,25,26].

**Figure 1:**
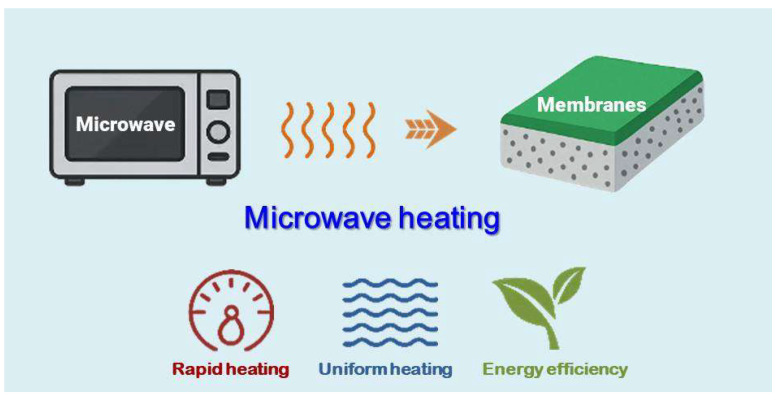
Membrane fabrication on a substrate via microwave synthesis.

Microwave heating is a rapid, uniform, and energy-efficient method that has gained increasing importance in material synthesis [27,28,29,30]. Since the 1990s, microwave synthesis has emerged and evolved as a novel method for producing zeolite materials [31,32]. Compared with conventional heating methods, microwave heating generates heat internally within the synthesis solution, leading to more uniform temperature distribution, faster heating rates, and greater overall efficiency [33,34,35,36]. This technique significantly reduces the synthesis time of zeolites while enhancing their crystallinity [37,38]. Moreover, due to its lower energy consumption, microwave heating is considered highly attractive from the perspective of green chemistry [39,40]. In recent decades, researchers have extended the use of microwave synthesis from zeolite powder production to the fabrication of zeolite and MOF membranes [41,42,43,44], membrane layers are typically formed on a substrate under microwave irradiation, as illustrated in Figure 1. Traditional synthesis methods for these membranes typically require long reaction times and substantial energy input. In contrast, microwave synthesis offers the key advantage of drastically reducing crystallization time, which has sparked growing interest. As a result, many researchers have actively explored and adopted this technique for the efficient fabrication of zeolite and MOF membranes [45,46,47,48]. However, most studies have primarily focused on exploiting the rapid synthesis capability, with comparatively little attention to optimizing the microwave heating process. Conventional microwave reactors typically operate at constant power, making the process highly sensitive to the reaction system and external conditions, and thereby complicating control of the crystallization process during membrane fabrication. Further research is therefore required to optimize microwave synthesis, which would allow its advantages to be more fully utilized and support the fabrication of high-performance membranes.

This article provides a brief overview of recent advances in the microwave synthesis of zeolite and MOF membranes, focusing on fundamental research and laboratory-scale studies, with a particular focus on optimizing the microwave heating process to support the continued development and broader application of this technique in membrane fabrication.

## 2. Fundamentals of Microwave Heating Technology

Microwave heating technology is based on the interaction between electromagnetic radiation and materials, generating heat uniformly throughout the entire volume of the material rather than relying on conventional surface-to-core heat transfer mechanisms [49,50]. Microwaves occupy the region of the electromagnetic spectrum between infrared and radio waves, with wavelengths ranging from 0.001 to 1 m and frequencies between 0.3 and 300 GHz [51,52]. For both laboratory and industrial applications, the most commonly used frequency is 2450 MHz, at which point microwave energy absorption by polar molecules, such as water, is maximized [53,54,55].

The heating effect of microwaves primarily arises from two mechanisms: dipole rotation and ionic conduction, through which microwave energy is converted into heat [35,56,57,58,59]. When exposed to the alternating electromagnetic field generated by microwaves, polar molecules within the material continuously attempt to realign themselves with the rapidly oscillating electric field. At the commonly used microwave frequency of 2.45 GHz, the electric field reverses direction approximately 4.9 billion times per second [60,61]. This constant reorientation of dipolar molecules, such as water, alcohols, and other polar species, leads to molecular friction and collisions, efficiently transforming electromagnetic energy into thermal energy. As a result, rapid and uniform heating is achieved throughout the material [59]. In addition to dipole rotation, ionic conduction also contributes to microwave heating when free ions or charged species are present, such as in salt solutions or precursor gels [62]. Under the oscillating electric field, these ions move back and forth, colliding with surrounding molecules. These collisions generate heat due to electrical resistance, further enhancing the overall heating effect [63]. Ionic conduction is particularly significant in systems containing dissolved salts, metal ions, or ionic liquids [62,64].

**Figure 2:**
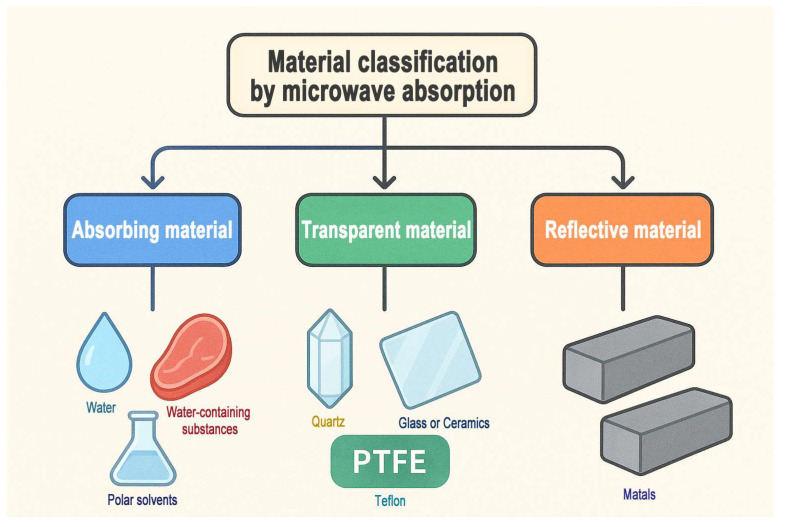
Classification of materials based on their ability to absorb microwave radiation.

The amount of heat generated per unit volume under microwave irradiation depends on the strength of the electric field, the frequency of the applied microwaves, and the dielectric properties of the material [49,58,65]. Notably, the generated heat is closely related to the dielectric constant (ε) and the dielectric loss factor (ε_r_″), with both factors playing a significant role in determining the heat generation [45,66]. Materials with higher dielectric loss factors can absorb microwave energy more effectively, resulting in improved heating performance in a microwave field [66,67,68]. Water and all water-containing substances exhibit high dielectric loss factors, making them excellent absorbers of both high-frequency and microwave energy [69,70]. The dielectric loss factors of water and several commonly used solvents are presented in Table 1. Generally, based on their ability to absorb microwave radiation, materials can be categorized into three types (Figure 2). Absorbing materials, such as water (ε_r_″ ≈ 12 at room temperature [71]), water-containing substances (including nearly all food products), and polar solvents, which can be efficiently heated under microwave irradiation. Transparent materials, such as quartz, glass, ceramics (ε_r_″ ≈ 0.0023 at room temperature [72]), and polytetrafluoroethylene (PTFE, Teflon), which allow microwaves to pass through with minimal absorption. Reflective materials, such as metals, which reflect most of the incident microwave energy and therefore do not undergo significant heating [45]. Importantly, within composite materials, specific components, such as polar solvents or chemical precursors, can selectively absorb microwave energy [69,73]. This selective absorption can lead to the formation of localized “hot spots,” where temperatures are significantly higher than the surrounding bulk environment [35,36]. These hot spots enhance reaction kinetics, promoting faster nucleation and crystal growth, which is particularly advantageous in material synthesis [35].

In summary, microwave synthesis offers several advantages over conventional heating methods, including more rapid and uniform temperature increases and minimized thermal gradients within the reaction system. The unique heating mechanisms of microwaves play a critical role in significantly shortening crystallization times and enhancing crystallinity. These benefits make microwave synthesis a highly promising and efficient strategy for the fabrication of zeolite and MOF membranes.

## 3. Typical Features and Benefits of Microwave Synthesis for Membrane Fabrication

**Figure 3:**
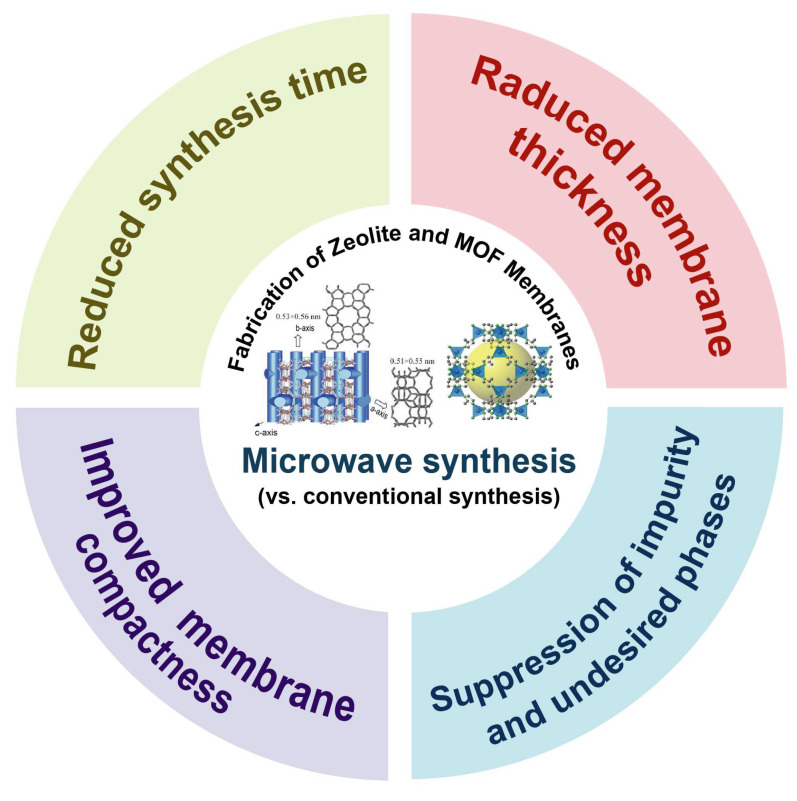
Typical features and benefits of microwave synthesis for membrane fabrication.

During the last few decades, microwave synthesis has exhibited several notable advantages over conventional approaches in the fabrication of both zeolite and MOF membranes [39]. As illustrated in Figure 3, these advantages include substantially shortened synthesis times, reduced membrane thickness, effective suppression of impurities and undesired phases, and enhanced membrane compactness. The key benefits and representative studies highlighting these improvements are summarized below:

### 3.1. Reduced Synthesis Time

One of the most significant advantages of microwave synthesis in zeolite membrane fabrication is the substantial reduction in synthesis time compared to conventional hydrothermal methods. Traditional hydrothermal synthesis often requires several hours or even days to achieve sufficient nucleation and crystal growth, largely due to the slow and non-uniform heat transfer from external heating sources to the reaction medium. In contrast, microwave heating delivers rapid, volumetric, and selective energy directly to the reaction mixture, enabling uniform temperature elevation within seconds to minutes. This accelerated and homogeneous heating promotes rapid supersaturation, thereby enhancing nucleation rates and crystal growth kinetics, and ultimately leading to a much shorter overall synthesis process.

**Figure 4:**
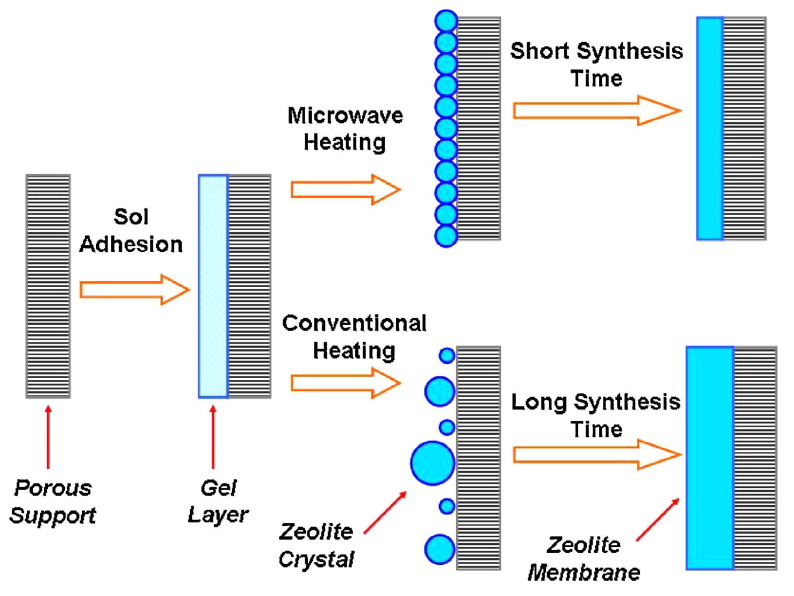
Comparative of different synthesis model for NaA zeolite membranes: microwave heating (15 min) vs. conventional heating (3 h). Reproduced with permission [45,76]. Copyright 2001, Elsevier.

Numerous studies have demonstrated the effectiveness of microwave synthesis in significantly reducing the time required for zeolite membrane fabrication [75,76,77,78]. For example, Yang et al. [75,76] investigated the synthesis of NaA zeolite membranes on porous α-Al_2_O_3_ supports using microwave synthesis methods (modified domestic microwave oven, microwave power not reported). In their study, dense and well-intergrown NaA zeolite membranes were successfully fabricated within just 15 min, in contrast to conventional hydrothermal methods, which typically require a minimum of 3 h to achieve comparable membrane quality [76]. The membranes were applied in gas separation, exhibiting similar H_2_/n-C_4_H_10_ permselectivities to those obtained by conventional heating, while the H_2_ permeance was approximately four times higher with microwave heating. To highlight this difference, a comparative synthesis model of NaA zeolite membranes prepared by microwave and conventional heating is presented in Figure 4. These findings clearly underscore the remarkable efficiency of microwave synthesis in accelerating zeolite membrane formation.

**Figure 5:**
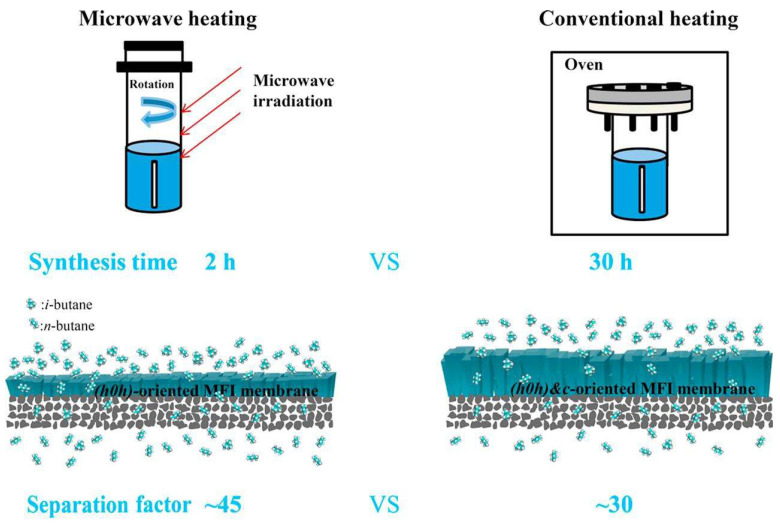
Schematic illustration of silicalite-1 membrane synthesis and separation performance: microwave heating (2 h, n-butane/i-butane = 45) vs. conventional heating (30 h, n-butane/i-butane = 32). Reproduced with permission [77]. Copyright 2019, Elsevier.

Similarly, Zhou and co-workers [77] reported the rapid fabrication of highly (h0h)-oriented silicalite-1 membranes on α-Al_2_O_3_ tubular supports using microwave synthesis (Mars microwave reaction system, CEM, Matthews, IN, USA, microwave power 600 W). In contrast to their earlier work employing conventional heating, where approximately 30 h were required to obtain selective silicalite-1 membranes, the microwave method reduced the synthesis time to just 2 h, representing a reduction of over 90%. Notably, the silicalite-1 membranes prepared via microwave synthesis not only exhibited significantly shorter fabrication times but also demonstrated enhanced separation performance, with the n-butane/i-butane separation factor increasing from approximately 32 to 45. Figure 5 is a schematic illustration of the synthesis time and separation performance of silicalite-1 membranes prepared by microwave and conventional heating. These findings further underscore the efficiency and performance advantages of microwave synthesis as a time-saving and effective strategy for zeolite membrane fabrication.

In addition to zeolite membranes, microwave synthesis has also been successfully applied to the rapid fabrication of MOF membranes [41,79,80,81,82]. Jeong et al. [79] reported the preparation of MOF-5 membranes on anodized aluminum oxide (AAO) substrates using microwave-assisted synthesis (Kenmore microwave equipment, Kenmore, Chicago, IL, USA, microwave power 500 W). The introduction of thin conductive layers, such as amorphous carbon or graphite, on the AAO substrates was found to significantly enhance heterogeneous nucleation and crystal growth kinetics under microwave conditions. As a result, well-packed MOF-5 membranes were obtained within just 20 to 30 s of microwave irradiation, a remarkable reduction in synthesis time compared to conventional solvothermal methods, which typically require several days to produce comparable membranes. Building on this work, Jeong et al. [80] further developed a secondary growth method to prepare continuous MOF-5 membranes with controllable out-of-plane orientation (Discover, CEM, Matthews, NC, USA, microwave power 300 W). This approach involved the rapid microwave-assisted deposition of densely packed MOF-5 seed layers, followed by solvothermal growth. The inclusion of a proton-scavenging amine was critical for preserving the seed crystals and promoting their growth. Preferentially oriented MOF-5 membranes were successfully obtained by initiating secondary growth from pre-oriented seed layers. This strategy underscores the versatility of microwave synthesis, not only in drastically reducing synthesis time but also in enabling structural and orientational control in MOF membrane fabrication. A similar rapid microwave approach has also been applied to the fabrication of other MOF membranes, which further demonstrates the potential of microwave methods in accelerating high-quality membrane synthesis [83,84,85,86].

The substantial reduction in synthesis time achieved through microwave synthesis offers considerable practical advantages for the fabrication of both zeolite and MOF membranes. Shortened synthesis durations not only improve production efficiency but also lower energy consumption, making the overall fabrication process more economical and environmentally sustainable.

### 3.2. Reduced Membrane Thickness

Microwave synthesis has been shown to effectively reduce the thickness of both zeolite and MOF membranes compared to conventional heating methods. Under microwave irradiation, the accelerated dissolution of reactants, such as precursor solutions or synthesis gels, leads to a rapid increase in nucleation density. The resulting abundance of nuclei quickly consumes the available reactants through fast crystallization, promoting the formation of small, uniformly sized crystals. This process suppresses excessive crystal growth and facilitates the development of a thin and uniform membrane layer.

**Figure 6:**
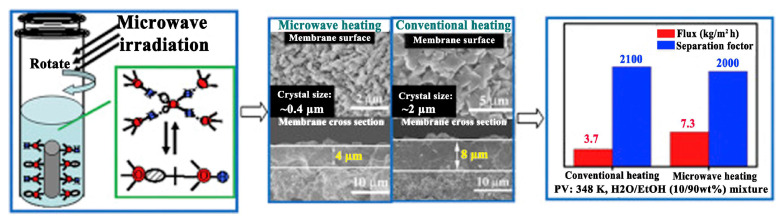
Schematic comparison of CHA zeolite membranes prepared by microwave and conventional heating: crystal size (0.4 μm vs. 2 μm), membrane thickness (4 μm vs. 8 μm), and ethanol dehydration separation performance (permeation flux: 7.3 kg·m^−2^·h^−1^ vs. 3.7 kg·m^−2^·h^−1^). Redrawn from Ref. [87]. Copyright 2016, Elsevier.

For zeolite membranes, Zhou et al. [87] reported the successful fabrication of chabazite (CHA) zeolite membranes with significantly smaller crystal sizes and reduced membrane thickness using microwave synthesis (MDS-10, Shanghai Sineo, Shanghai, China, microwave power 500 W) compared to conventional heating methods. Specifically, the CHA membranes synthesized via microwave irradiation exhibited crystal sizes of around 0.4 µm, about one-fifth of the 2 µm crystals produced using conventional heating, and membrane thickness around 4 µm, approximately 50% of those produced using conventional heating (8 µm). This notable reduction was attributed to the unique heating mechanism of microwave irradiation, which activates water molecules and enhances their ability to dissolve the synthesis gel. The resulting increased dissolution generates a higher concentration of nuclei, promoting rapid and uniform crystallization while effectively suppressing excessive crystal growth [73]. Consequently, thinner zeolite membranes composed of smaller, more uniform crystals were obtained. In ethanol dehydration, the permeation flux of microwave-prepared membranes reached 7.3 kg·m^−2^·h^−1^, nearly double that of membranes prepared by conventional methods (3.7 kg·m^−2^·h^−1^) (Figure 6). This structural refinement led to improved membrane performance, with the water flux of the microwave-synthesized membranes approximately doubling that of membranes prepared by conventional methods.

In another study, Chew et al. [88] successfully fabricated SAPO-34 membranes using microwave synthesis (MARS 5, CEM, Matthews, NC, USA, microwave power not reported) with a colloidal solution containing tetraethylammonium hydroxide as the structure-directing agent. The resulting membranes consisted of homogeneous SAPO-34 crystals with an average size of approximately 0.7 μm. Compared to conventional hydrothermal methods, microwave heating significantly narrowed the crystal size distribution, which was attributed to the uniform and efficient volumetric heating provided by microwave irradiation. Additionally, microwave synthesis effectively reduced the membrane thickness, yielding SAPO-34 membranes with thicknesses of only 1–2 μm, substantially thinner than the 3.6–5.5 μm typically observed in membranes prepared via conventional hydrothermal synthesis. Beyond thickness reduction, microwave synthesis also dramatically shortened the overall synthesis time, achieving complete membrane formation within just 2 h at 200 °C, in contrast to the 24 h required under conventional conditions.

Additionally, Sebastian et al. [42] reported the fabrication of MFI-type zeolite membranes on ceramic capillary supports using microwave synthesis (Milestone ETHOS 1600, Milestone, Sorisole, Italy, microwave power not reported). The resulting membranes exhibited a significantly reduced thickness of approximately 1.2 μm, in contrast to a thickness of 7.4 μm observed in membranes prepared under the same conditions using conventional heating. Notably, the thicker membranes produced via conventional heating contained a higher number of hydrophilic intercrystalline defects, which adversely affected separation performance by reducing the selective permeation of ethanol. In comparison, the thinner membranes synthesized using microwave irradiation not only minimized mass transfer resistance but also suppressed defect formation. As a result, these membranes demonstrated enhanced pervaporation flux and improved separation selectivity in the ethanol–water separation process. At 45 °C, the best performance achieved with the MFI membrane was a permeation flux of 1.5 kg/h·m^2^ and an ethanol/water selectivity of 54.

**Figure 7:**
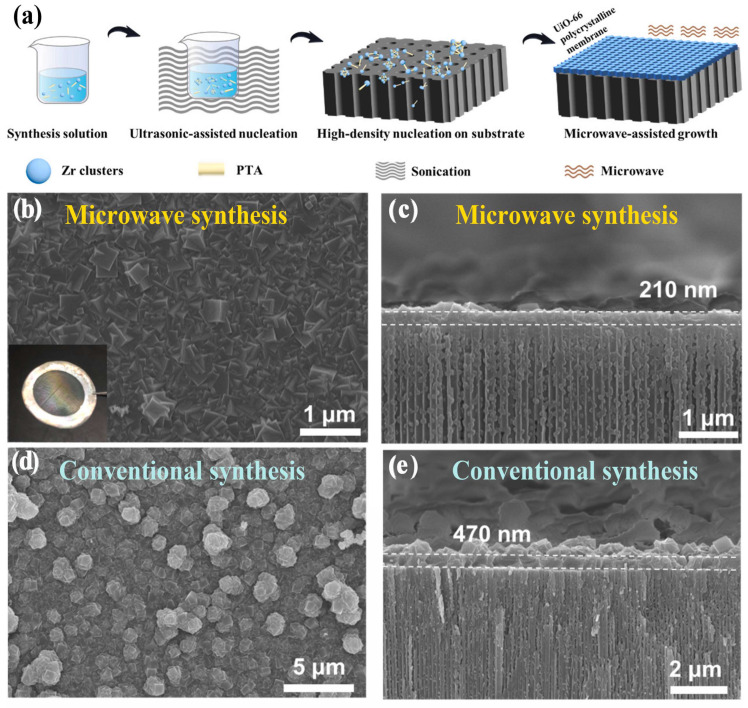
(**a**) Schematic illustration of the fabrication of UiO-66 membranes by microwave synthesis. (**b**–**e**) Surface and cross-sectional SEM images of UiO-66 membranes synthesized by microwave heating (1 h, ~210 nm) and conventional solvothermal treatment (130 °C, 24 h, ~470 nm). Adapted from Ref. [89]. Copyright 2022, Elsevier.

For MOF membranes, Wei et al. [89] reported the rapid fabrication of ultrathin UiO-66 polycrystalline membranes using a combined sonication–microwave synthesis strategy (flexiWAVE microwave equipment, Milestone, Sorisole, Italy, microwave power not reported). In this method, ultrasonication at room temperature was first employed to generate a high density of uniformly distributed nucleation sites on the substrate surface. This was followed by microwave synthesis, which facilitated the rapid intergrowth of these nuclei into a continuous and compact membrane layer. Figure 7a illustrates the preparation process of this method. As a result, uniform, defect-free UiO-66 membranes with a thickness of approximately ~210 nm were successfully synthesized within just 1 h. In contrast, when the same nucleation step was followed by conventional solvothermal treatment at 130 °C for 24 h, the resulting membrane exhibited a significantly greater thickness of around 470 nm and poorer surface flatness. Figure 7b–e displays the surface and cross-sectional SEM images of the UiO-66 membranes obtained by microwave synthesis and by conventional solvothermal treatment, as reported by Wei et al. [89]. These results clearly demonstrate that, compared to conventional methods, microwave synthesis not only significantly shortens synthesis time but also enables the formation of thinner MOF membranes with improved surface quality. In terms of separation performance, the microwave-synthesized UiO-66 membranes exhibited excellent metal ion rejection, achieving a Na^+^ rejection of 99.6% with a permeation rate of 0.0005 mol·m^−2^·h^−1^ sustained for over 700 h, together with a forward osmosis water flux of 0.16 L·m^−2^·h^−1^·bar^−1^.

As a result of the rapid and uniform heating enabled by microwave synthesis, both zeolite and MOF membranes typically exhibit significantly reduced thickness compared to those prepared using conventional methods. Thinner membrane layers contribute to lower mass transfer resistance and increased permeation flux. Thus, microwave synthesis not only accelerates membrane fabrication but also supports the development of high-performance membranes with enhanced transport efficiency for use in separation processes.

### 3.3. Suppression of Impurity and Undesired Phases

Microwave synthesis provides significant advantages in minimizing the formation of impurities and undesired crystalline phases during membrane fabrication, primarily due to its rapid and uniform volumetric heating. In zeolite synthesis, nucleation and crystal growth are kinetically driven and highly sensitive to temperature fluctuations. Conventional heating methods, characterized by slow and uneven heat transfer, often lead to prolonged overlap between the nucleation and growth stages, making precise control over phase evolution challenging. In contrast, microwave irradiation enables rapid temperature ramp-up, allowing the reaction system to reach the desired temperature in a much shorter time. This swift heating narrows the temporal window for nucleation, thereby reducing the likelihood of uncontrolled bulk nucleation. As a result, the crystallization process becomes more controlled, significantly lowering the tendency for the formation of twin crystals, impurities, and non-target phases within the membrane layer.

A notable study that illustrates the advantage of microwave synthesis in reducing the formation of impurities and non-target phases is the work by Coutinho et al. on ETS-4 zeolites and ETS-4 thin films (MARSXpress™, CEM, Matthews, NC, USA, microwave power ≤ 300 W) [90]. In this research, microwave heating not only significantly accelerated the crystallization process but also enhanced phase purity. Pure ETS-4, free of any detectable impurities, was successfully synthesized within just 1 h using microwave irradiation. This represents a substantial improvement over conventional hydrothermal methods, which typically require 36 to 48 h and often lead to the formation of undesired phases such as GTS-1 or analcime. These findings underscore the ability of microwave synthesis to suppress the formation of unwanted crystalline phases through rapid and uniform heating, thereby offering an effective strategy for improving crystal phase purity in membrane fabrication.

Another compelling example of the advantages of microwave synthesis in improving phase purity is the study by Xu et al., who successfully fabricated a high-purity hydroxy-sodalite zeolite membrane on an α-alumina support using microwave-assisted synthesis (modified domestic microwave oven, microwave power not reported) [91]. In contrast to conventional hydrothermal methods, which typically yielded mixed-phase membranes containing NaX, NaA, and hydroxy-sodalite zeolites, the microwave approach produced a phase-pure hydroxy-sodalite membrane in just 45 min, representing a more than eightfold reduction in synthesis time. This improvement was attributed to the rapid and homogeneous heating provided by microwave irradiation, which facilitated the swift dissolution of the precursor gel at the support surface and enabled the simultaneous nucleation of uniformly sized hydroxy-sodalite crystals. In comparison, conventional hydrothermal synthesis suffered from asynchronous nucleation due to slower and less uniform heat transfer, leading to non-uniform crystal sizes and the formation of impurity phases. As a result, the microwave-synthesized membrane not only exhibited superior phase purity but also demonstrated excellent gas separation performance, with a hydrogen/n-butane permselectivity exceeding 1000. These findings further underscore the potential of microwave synthesis to enhance crystal phase purity through improved thermal control and crystallization kinetics, offering more precise regulation of membrane formation and superior separation performance.

**Figure 8:**
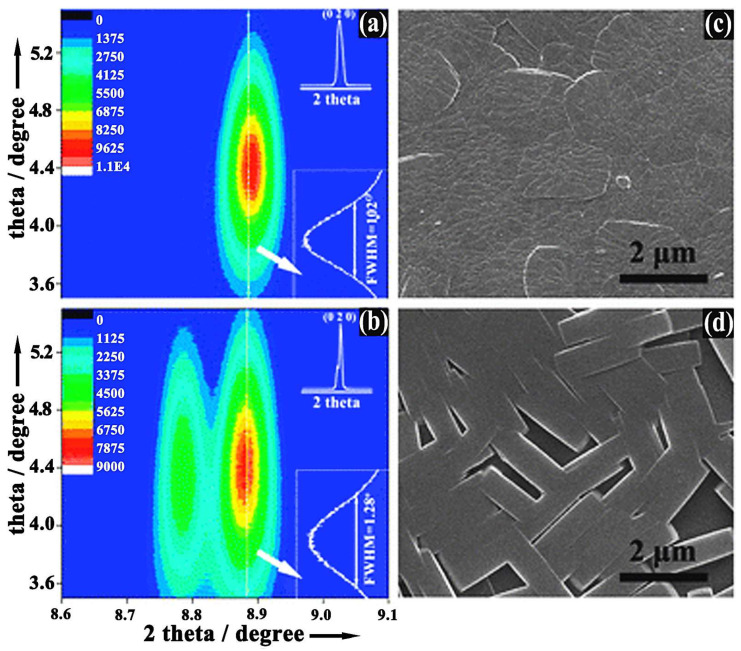
Comparison of *b*-oriented MFI zeolite films prepared by (**a**,**c**) microwave and (**b**,**d**) conventional synthesis: (**a**,**b**) X-ray rocking curve analysis; (**c**,**d**) SEM images of membrane surfaces. Redrawn from Ref. [92]. Copyright 2012, Royal Society of Chemistry.

Liu and co-workers reported the rapid fabrication of highly *b*-oriented MFI zeolite films without the presence of undesired *a*-oriented twins using microwave synthesis (MICROSYNTH, Milestone, Sorisole, Italy, microwave power ≤ 300 W) [92]. Figure 8 presents the characterization results of the *b*-oriented MFI zeolite films prepared by microwave synthesis and conventional hydrothermal methods. The study demonstrated that microwave irradiation effectively suppressed the growth of *a*-oriented twin domains during the formation of *b*-oriented MFI films, in contrast to conventional hydrothermal methods. X-ray rocking curve analysis further confirmed that the *b*-oriented MFI grains synthesized via microwave heating exhibited higher structural regularity and orientation precision compared to those produced by traditional approaches. Electrochemical oxidation tests conducted using MFI film–modified Pt electrodes revealed that the suppression of twin growth significantly enhanced the diffusion rate of methanol molecules through the zeolite nanochannels. These findings underscore the capability of microwave synthesis to precisely regulate the crystallization process and control crystal orientation, thereby enabling the fabrication of well-oriented and high-performance zeolite membranes.

Márquez and co-workers reported the successful synthesis of MIL-100 nanoparticles using microwave irradiation under mild reaction conditions (Mars, CEM, Matthews, IN, USA, microwave power 400–1600 W) [93]. These nanoparticles were subsequently employed to fabricate thin films via a dip-coating technique. Notably, in comparison to conventional synthesis methods, the microwave-assisted approach efficiently produced phase-pure MIL-100 without any detectable MIL-96 impurities. This result highlights the effectiveness of microwave synthesis in suppressing impurity formation and preventing undesired phase transitions during MOF material fabrication. Furthermore, several other studies have also demonstrated the successful synthesis of phase-pure MOF membranes using microwave-assisted methods [94,95,96], thereby reinforcing the reliability and versatility of this technique in the production of high-purity MOF membranes.

Microwave heating has demonstrated a remarkable capability to suppress the formation of impurities and undesired crystalline phases during membrane synthesis. The rapid and volumetric energy transfer inherent to microwave irradiation promotes uniform nucleation and accelerates crystallization kinetics, thereby narrowing the time window available for secondary phase formation. Consequently, membranes with higher phase purity and more uniform structures can be achieved. This improved control over phase evolution facilitates more precise regulation of the synthesis process and supports the selective formation of the desired membrane phase. 

### 3.4. Improved Membrane Compactness

Microwave synthesis also holds significant promise for improving membrane compactness. The rapid, uniform, and energy-efficient heating characteristic of microwave irradiation facilitates the formation of small crystals with narrow size distributions and reduces the temporal overlap between nucleation and crystal growth stages, promoting their effective separation during crystallization [39,73]. These effects contribute to a reduction in intercrystalline voids and support the development of denser and more compact membrane structures. The following representative studies highlight the application of microwave synthesis in enhancing the compactness of both zeolite and MOF membranes.

Wang et al. successfully synthesized continuous *b*-oriented MFI membranes on stainless steel substrates using microwave synthesis (microwave type and microwave power not reported) [97]. In this process, a large number of nuclei were first formed on the support surface through microwave-assisted aging at relatively low temperatures. During the subsequent microwave crystallization stage, these nuclei rapidly grew into submicron-sized zeolite crystals. As the crystals enlarged, the degree of intergrowth between adjacent grains improved, ultimately resulting in the formation of a dense and continuous *b*-oriented MFI membrane. Compared to conventional heating methods, the microwave-assisted approach significantly accelerated crystal growth, enhanced membrane compactness, and substantially reduced overall crystallization time.

Li et al. reported that microwave synthesis (domestic microwave oven, microwave power not reported) could produce LTA membranes with significantly improved compactness compared to those fabricated using conventional heating methods [98]. While SEM images suggested that membranes from both approaches appeared similarly compact, the authors employed pervaporation tests to more precisely assess membrane integrity. This evaluation leveraged the strong hydrophilicity of LTA zeolites, which causes water to preferentially adsorb not only in zeolitic pores but also in non-zeolitic voids. Such preferential adsorption effectively blocks the permeation of less polar molecules like isopropanol. However, at low water concentrations, this blocking effect diminishes, allowing isopropanol to traverse larger non-zeolitic pores and thereby reducing overall separation selectivity. Both types of membranes exhibited high dehydration performance when separating feed mixtures containing 95.0% isopropanol. However, when the water content dropped below 2.0%, the conventionally synthesized membrane lost its separation capability, whereas the microwave-synthesized membrane maintained effective separation. This contrast was attributed to the presence of nanoscale non-zeolitic pores in the conventionally synthesized membrane, which were significantly suppressed in the membrane produced via microwave irradiation. The rapid and uniform heating provided by microwave synthesis was found to promote more compact membrane structures with fewer defects. These findings were further corroborated by the authors’ investigation of FAU-type zeolite membranes, where microwave synthesis similarly enhanced membrane compactness [45]. As a result, the pervaporation selectivity of Y-type zeolite membranes for ethanol/water mixtures was noticeably improved through microwave-assisted fabrication. Recently, Gordina et al. [99] demonstrated that LTA membranes synthesized by microwave heating exhibited high compactness (microwave type not reported, microwave power 600 W), an absence of non-zeolite pores, and excellent water/ethanol pervaporation performance at 60 °C. The separation coefficient of the microwave-synthesized membranes reached 9949, while that of conventionally synthesized membranes was below 360 under the same conditions.

**Figure 9:**
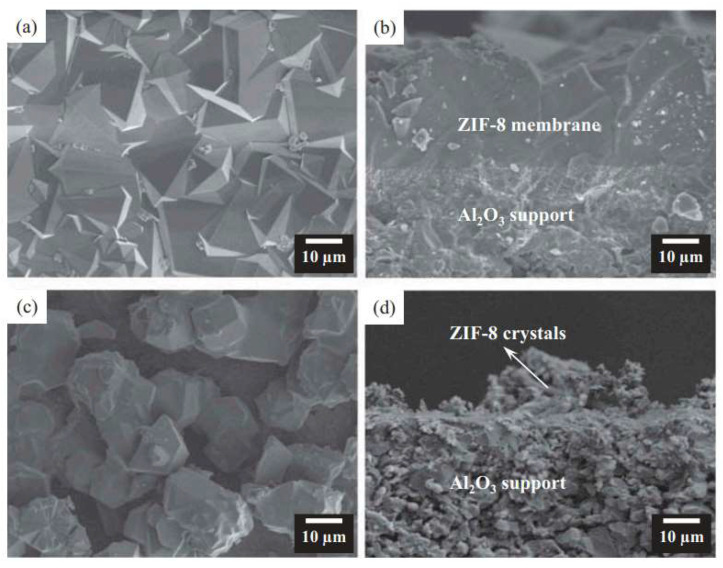
Surface and cross-sectional SEM images of ZIF-8 membranes synthesized by (**a**,**b**) microwave heating and (**c**,**d**) conventional solvothermal heating. Reproduced with permission [100]. Copyright 2016, Taylor & Francis Group.

Huang and co-workers demonstrated that phase-pure and compact ZIF-8 membranes could be successfully fabricated on relatively coarse supports using microwave synthesis (ETHOS One, Milestone, Sorisole, Italy, microwave power not reported) [100]. Figure 9 presents the surface and cross-sectional morphologies of ZIF-8 membranes prepared by microwave synthesis and by conventional solvothermal methods. In their study, the resulting membranes featured a continuous surface layer composed of densely packed rhombic dodecahedral ZIF-8 crystals, free of visible cracks, pinholes, or other structural defects. This well-intergrown crystal arrangement formed a uniform and compact membrane layer. In contrast, membranes prepared via conventional solvothermal heating exhibited only sparse and isolated ZIF-8 crystals on the surface, indicating that crystal nucleation and growth were less effective under traditional heating conditions. Compared to conventional methods, microwave synthesis offers several advantages, including significantly reduced synthesis time and the formation of finer, more uniformly distributed crystals. The rapid and homogeneous heating provided by microwave irradiation accelerates the generation of numerous ZIF-8 nuclei on the support surface, thereby promoting dense crystal packing and improving membrane compactness. In terms of separation performance, the microwave-synthesized ZIF-8 membranes exhibited high CO_2_/CH_4_ selectivity (separation factor of 6.8 for an equimolar mixture at 100 °C, 2 bar feed pressure, and 1 bar permeate pressure) and a CO_2_ permeance of 1.02 × 10^−8^ mol·m^−2^·s^−1^·Pa^−1^, with stable performance during long-term operation.

Moreover, the enhanced intergrowth between crystals contributes to the elimination of structural defects and intercrystalline voids. As a result, microwave-synthesized membranes tend to exhibit superior structural integrity and improved separation performance.

## 4. Latest Developments in Microwave Heating Process Optimization

Microwave systems offer distinct advantages in delivering rapid and uniform energy, enabling high heating rates and localized thermal effects that are difficult to achieve with conventional heating methods [39,70]. Traditionally, microwave-assisted membrane synthesis has been performed under multi-mode conditions using fixed power settings to rapidly elevate temperature, resulting in high-quality membranes within significantly shorter synthesis times. While this approach has demonstrated clear benefits over conventional heating, most studies have focused primarily on using microwave energy for rapid synthesis, with relatively limited emphasis on optimizing the microwave heating process itself.

**Figure 10:**
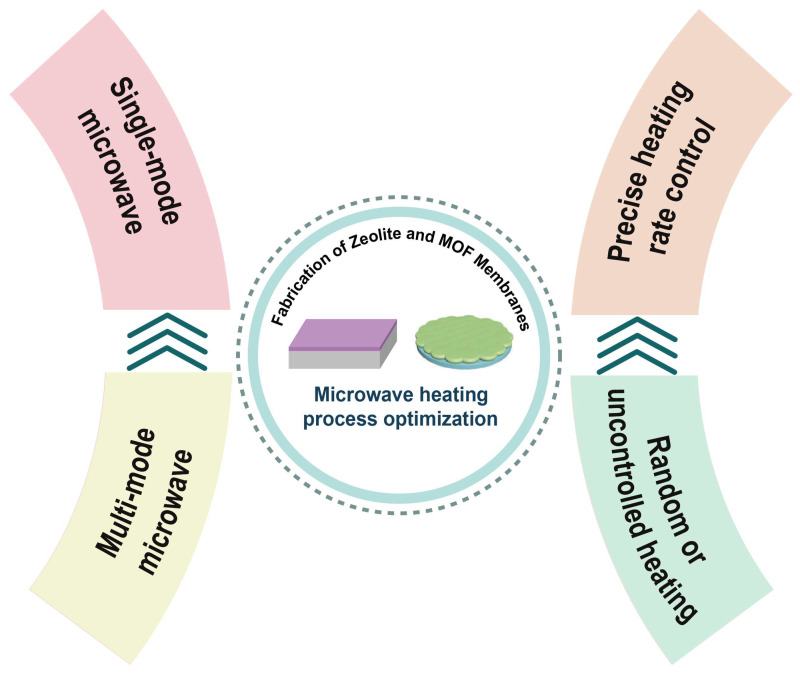
The latest developments in microwave heating process optimization for membrane preparation, highlighting two major strategies: single-mode microwave and precise heating rate control.

In recent years, however, single-mode microwave heating and precise heating rate control have emerged as advanced strategies aimed at enhancing the control and tunability of membrane fabrication. As illustrated in Figure 10, these approaches represent the latest developments in microwave process optimization, offering improved precision over thermal conditions and crystal growth dynamics. The following sections review recent progress in this area, particularly the implementation of single-mode microwave systems and the regulation of heating rates, both of which have shown significant promise in enhancing membrane quality and performance.

### 4.1. Single-Mode Microwave

Microwave reactors used for membrane synthesis are broadly classified into multi-mode and single-mode systems, based on the nature of electromagnetic field propagation within the cavity. Multi-mode cavities, typically larger in volume and capable of supporting multiple resonant modes, often produce non-uniform field distributions due to wave reflections and mode interference. In contrast, single-mode microwave reactors operate with a single, well-defined electromagnetic mode within a smaller cavity, resulting in a more uniform and concentrated energy distribution [39,70]. As a result, single-mode systems typically achieve power densities one to two orders of magnitude higher than those of multi-mode configurations. These differences significantly affect the thermal environment during synthesis, thereby influencing nucleation and crystal growth kinetics, and ultimately determining the membrane’s microstructure and separation performance in practical applications.

**Figure 11:**
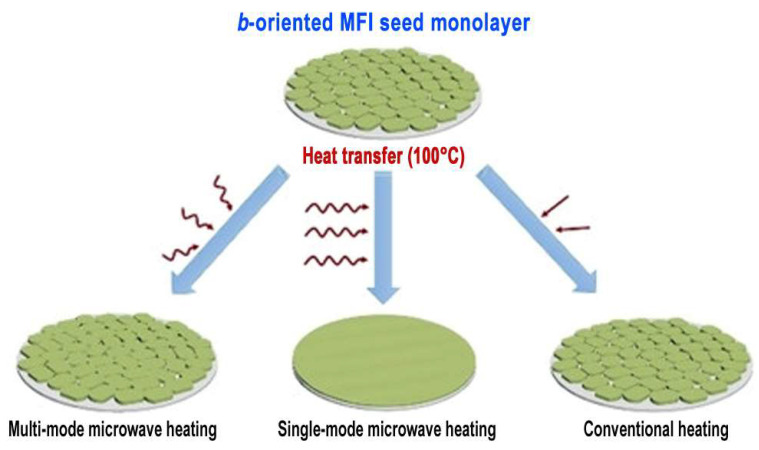
Schematic illustration of *b*-oriented MFI membrane fabrication by (**right**) conventional heating, (**left**) multi-mode microwave synthesis, and (**middle**) single-mode microwave synthesis. Reproduced with permission [101]. Copyright 2020, Wiley.

Liu and co-workers successfully demonstrated the use of single-mode microwave heating in the synthesis of both zeolite and MOF membranes (Discover, CEM, Matthews, NC, USA, microwave power not reported) [101,102]. In a representative study on MFI zeolite membranes, a compact and uniformly aligned *b*-oriented MFI seed layer was first deposited onto a flat substrate via manual rubbing. Subsequent epitaxial growth under single-mode microwave irradiation produced well-intergrown and strongly *b*-oriented MFI films with minimal twin defects [101]. Compared to conventional multi-mode microwave systems, the superior field uniformity and higher energy density of single-mode irradiation enabled simultaneous suppression of out-of-plane twin growth and promotion of in-plane epitaxial growth, as illustrated in Figure 11. These effects were achieved under mild synthesis conditions (≤100 °C, 2 h), and the resulting membranes exhibited precise molecular sieving behavior. Notably, the same group previously applied single-mode microwave heating to the fabrication of MOF membranes. In their work on NH_2_-MIL-125(Ti), a dynamic air–liquid interface-assisted self-assembly technique was used to construct a highly *c*-oriented seed monolayer, followed by in-plane epitaxial growth using single-mode microwave irradiation [102]. The controlled microwave field was crucial in maintaining crystal orientation and suppressing twin formation. The resulting membranes exhibited significantly enhanced H_2_/CO_2_ selectivity of 24.8, over six times higher than that of randomly oriented membranes, underscoring the importance of orientation control in optimizing separation performance. Liu and co-workers applied single-mode microwave synthesis to the fabrication of other zeolite and MOF membranes [103,104,105,106], demonstrating its feasibility and effectiveness in optimizing membrane microstructures. Together, these studies highlight the potential of single-mode microwave systems to enhance membrane microstructure and separation performance in zeolite and MOF membranes.

These studies suggest that single-mode microwave heating, due to its uniform and intense electromagnetic field characteristics, serves as an effective strategy for controlling membrane orientation and microstructure, thereby enhancing separation performance.

### 4.2. Precise Heating Rate Control

In conventional microwave-assisted membrane synthesis, heating is typically conducted at a fixed microwave power level, resulting in rapid but uncontrolled temperature increases. Under such conditions, the temperature profile tends to be unpredictable: the heating rate may be high initially but slow down over time, or it may fluctuate throughout the process. This variability stems from changes in environmental conditions and the dynamic heat generation and dissipation characteristics of the reaction system. Given that reaction temperature and duration critically influence crystal nucleation and growth, instability in the heating rate complicates precise control over the nucleation–growth balance. This lack of control poses a significant challenge to further improving membrane quality.

Recent advances in microwave reactor design and control strategies have enabled more precise regulation of heating rates through techniques such as programmable power modulation and feedback-based temperature control. These innovations allow for the implementation of tailored temperature profiles that sustain constant or specifically designed heating rates throughout the synthesis process. Such precise control over the heating rate facilitates better regulation of crystallization dynamics, particularly the balance between nucleation and growth, thereby enhancing membrane quality and separation performance in practical applications. Although only a limited number of studies have focused on this aspect, the following section reviews recent progress in the application of precise heating rate control in microwave-assisted membrane fabrication.

**Figure 12:**
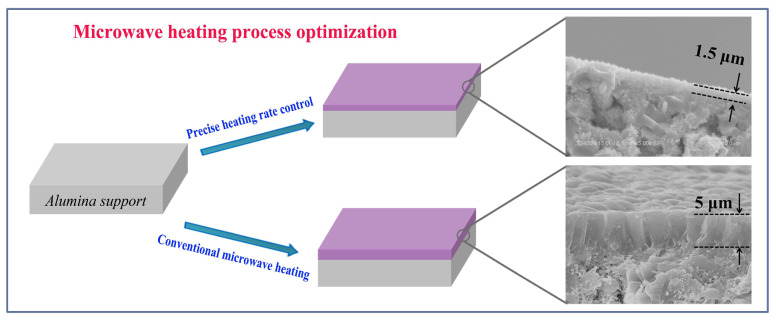
Schematic illustration of mordenite membrane thickness reduction, comparing conventional microwave synthesis (~5 μm) with microwave synthesis under precise heating rate control (~1.5 μm). Redrawn from Ref. [107]. Copyright 2020, Elsevier.

Li and co-workers investigated the influence of heating rate on the microstructure and performance of mordenite membranes synthesized via microwave heating (modified multifunctional microwave reactor based on MWave-5000, Shanghai Sineo, Shanghai, China, microwave power not reported) [107]. It is well known that temperature is a key factor in the crystallization of zeolite crystals [108]. It is generally accepted that lower temperatures favor nucleation, while higher temperatures promote crystal growth. In conventional hydrothermal synthesis, these two stages often overlap due to gradual heating, complicating precise control [2,39,109]. In contrast, the rapid and uniform heating characteristic of microwave systems minimizes this overlap, allowing partial separation of nucleation and growth. Leveraging this advantage, Li and colleagues developed a two-stage variable heating-rate protocol: a slow heating rate was applied at lower temperatures to ensure uniform nucleation, followed by a rapid heating phase to accelerate crystal growth. Compared with conventional microwave synthesis (modified domestic microwave oven, Shanghai Sineo, Shanghai, China, microwave power not reported) [110], this approach produced thinner, denser, and better-intergrown mordenite membranes while significantly reducing total crystallization time, from 4 h to 75 min, and membrane thickness, from approximately ~5 μm to ~1.5 μm, as illustrated in Figure 12, by conventional microwave synthesis and microwave synthesis with precise heating rate control, respectively. The optimized membranes also exhibited superior pervaporation performance for the dehydration of 90 wt% acetic acid/water mixtures at 75 °C, with permeation flux increasing by over 70% (from 0.87 to 1.48 kg.m^−2^ h^−1^). Li and co-workers extended this precise heating-rate control strategy under microwave synthesis to the fabrication of high-performance ZSM-5 membranes [111]. In acetic acid dehydration, the membrane exhibited outstanding separation performance, achieving a high permeation flux of 1.87 kg·m^−2^ h^−1^ and a separation factor exceeding 10,000. This study demonstrates that precise control of the heating rate in microwave synthesis is a promising strategy for enhancing membrane fabrication, enabling reductions in synthesis time and membrane thickness, alongside significant improvements in separation performance.

In summary, although research on precise heating rate control in microwave-assisted membrane synthesis remains in its early stages, it shows considerable potential for enhancing membrane quality. Excessively rapid heating may increase the randomness of crystal growth, leading to structural irregularities and reduced membrane performance. In contrast, controlled and moderate heating rates enable a more balanced progression of nucleation and crystal growth, resulting in improved membrane microstructure, reduced synthesis time, and enhanced separation efficiency. This level of control is especially valuable in microwave systems, where rapid and uniform energy delivery can be strategically harnessed to optimize crystallization dynamics. Continued exploration of tailored heating profiles is expected to further advance the fabrication of high-performance membranes.

## 5. Summary and Outlook

Microwave is a rapid, uniform, and energy-efficient heating method. Microwave synthesis has demonstrated significant advantages over conventional methods for the preparation of zeolite and MOF membranes, including shorter synthesis times, reduced membrane thickness, suppression of impurity phases, and enhanced membrane compactness. These benefits stem from the rapid and uniform heating characteristics of microwave synthesis, which accelerates crystallization and improves membrane quality. Despite its advantage in reducing reaction times, the rapid heating inherent to microwave synthesis can lead to the heating stage being overlooked. Such oversight may impede the formation of high-performance membranes and complicate process control during membrane fabrication. To date, most studies have focused on the rapid synthesis capability of microwave heating, with comparatively little attention given to optimizing the heating process itself.

Recent advancements in microwave process optimization, particularly the use of single-mode microwave systems and precise heating rate control, offer promising avenues for further improving membrane microstructure and separation performance. Single-mode systems provide more uniform and intense electromagnetic fields by separating the electric and magnetic components of the microwaves, allowing materials that couple preferentially with either field to absorb energy more efficiently. This targeted energy delivery promotes better crystal orientation and suppresses defects. During the heating process, precise control of heating rates allows for finer regulation of nucleation and crystal growth, facilitating the formation of thinner, denser, and more uniform membranes. The development of programmable and feedback-controlled microwave reactors is expected to enable tailored thermal profiles, allowing more precise tuning of crystallization kinetics. Although research on heating rate control is still limited, its technical feasibility and demonstrated benefits make it a compelling focus for future work. As microwave technologies and control strategies continue to evolve, microwave process optimization is poised to play a key role in advancing the fabrication of high-performance membranes.

Nevertheless, several challenges remain for broader implementation. The extremely rapid heating and drastically shortened synthesis time demand precise control of the heating process and high reactor performance. Most commercial microwave reactors lack single-mode systems or dedicated modules for heating-rate control due to the complexity involved in designing and developing these components. Implementing these functions often requires costly modifications or custom designs. Although many researchers recognize the potential benefits of such process optimization, high equipment costs and the additional time needed for system upgrades or retrofitting can impede broader adoption. Moreover, the synthesis of certain zeolite and MOF membranes on long-tube substrates for future industrial-scale microwave-assisted applications frequently demands high-temperature and high-pressure conditions, placing stringent requirements on the autoclave chambers of microwave reactors. Unlike conventional autoclaves, stainless steel vessels cannot be used, as they block microwave penetration. Typical wave-transparent materials, such as Teflon, are therefore used to fabricate long-tube autoclaves for membrane growth on long-tube substrates. However, producing these Teflon-lined autoclaves in such specialized forms is complex, and maintaining a stable crystallization environment under high-temperature and high-pressure conditions remains a significant challenge. Overcoming these technical and material challenges will be essential to fully realize the advantages of microwave-assisted membrane synthesis and to facilitate the transition from laboratory-scale research to industrial-scale applications.

## Figures and Tables

**Table 1:** Dielectric loss factors of water and commonly used solvents at 2.45 GHz and 20 °C.

No.	Solvent	Dielectric Loss Factors (εr″)	Reference
1	Water (H_2_O)	~12	[71]
2	Dimethyl sulfoxide (DMSO)	~12.5	[71]
3	Ethanol (EtOH)	6.46	[66]
4	Methanol (MeOH)	11.77	[66]
5	Propanol (PrOH)	3.41	[66]
6	n-Butanol (n-BuOH)	1.45	[66]
7	1-Pentanol (1-PentOH)	~1.1	[74]

## Data Availability

No new data were created.
